# Deficiency in Serine Protease Inhibitor Neuroserpin Exacerbates Ischemic Brain Injury by Increased Postischemic Inflammation

**DOI:** 10.1371/journal.pone.0063118

**Published:** 2013-05-03

**Authors:** Mathias Gelderblom, Melanie Neumann, Peter Ludewig, Christian Bernreuther, Susanne Krasemann, Priyadharshini Arunachalam, Christian Gerloff, Markus Glatzel, Tim Magnus

**Affiliations:** 1 Department of Neurology, University Medical Center Hamburg-Eppendorf, Hamburg, Germany; 2 Institute of Neuropathology, University Medical Center Hamburg-Eppendorf, Hamburg, Germany; University of Cologne, Germany

## Abstract

The only approved pharmacological treatment for ischemic stroke is intravenous administration of plasminogen activator (tPA) to re-canalize the occluded cerebral vessel. Not only reperfusion but also tPA itself can induce an inflammatory response. Microglia are the innate immune cells of the central nervous system and the first immune cells to become activated in stroke. Neuroserpin, an endogenous inhibitor of tPA, is up-regulated following cerebral ischemia. To examine neuroserpin-dependent mechanisms of neuroprotection in stroke, we studied neuroserpin deficient (*Ns^−/−^*) mice in an animal model of temporal focal ischemic stroke. Infarct size and neurological outcome were worse in neuroserpin deficient mice even though the fibrinolytic activity in the ischemic brain was increased. The increased infarct size was paralleled by a selective increase in proinflammatory microglia activation in *Ns^−/−^* mice. Our results show excessive microglial activation in *Ns^−/−^* mice mediated by an increased activity of tPA. This activation results in a worse outcome further underscoring the potential detrimental proinflammatory effects of tPA.

## Introduction

The therapeutic administration of plasminogen activator (tPA) in cerebral ischemia aims at the re-canalization of the occluded vessel [1]. In clinical studies modest but significant improvements in clinical outcome have been observed in patients treated with intravenous recombinant tPA up to 4.5 h after symptom onset [2].

tPA is a serine protease that converts plasminogen to plasmin leading to subsequent fibrin degradation. Despite the unquestionable beneficial tPA effects in clinical studies, animal studies revealed that endogenously produced or exogenously administered tPA has multiple roles in stroke pathology including plasminogen dependent beneficial and plasminogen dependent and independent deleterious effects [3]–[5]. Beside its beneficial thrombolytic activity in the vascular compartment, tPA contributes to an ischemia induced increase in permeability of the neurovascular unit at the blood brain interface with subsequent development of harmful brain edema [5]. In the brain parenchyma, tPA aggravates excitotoxic neuronal cell death via interaction with *N*-metyl-*D*-aspartate (NMDA) receptors, degradation of components of the extracellular matrix, activation of microglial cells, and induction of pro-inflammatory cytokines (for review see [4]).

A molecule inhibiting adverse tPA effects in stroke is neuroserpin, a serine protease inhibitor specific to the brain [6]. Neuroserpin belongs to the superfamily of serpins and plays an important role in brain development, neuronal survival, and synaptic plasticity [5]. Its only known substrate is tPA [7].

In animal models of ischemic stroke overexpression of neuroserpin and treatment with neuroserpin led to a decrease in infarct size [5], [8]. Moreover, decreased levels of neuroserpin following stroke correlated with brain damage and polymorphisms in the neuroserpin gene may be associated with the risk of early onset stroke in Caucasian women [9]–[11].

To further investigate the role of neuroserpin in stroke, we used neuroserpin-deficient mice, subjected them to temporary middle cerebral artery occlusion (MCAO) and studied their response to cerebral ischemia.

## Methods

### Animals

All animal experiments were approved by the local animal care committee (Behörde für Lebensmittelsicherheit und Veterinärwesen), conducted according to the recently published recommendations for research in mechanism-driven basic stroke studies [12] and were performed in accordance with the ARRIVE guidelines (http://www.nc3rs.org/ARRIVE).

To minimize animal suffering dropout criteria were defined. Animals, which fulfilled dropout scores were euthanized with isoflurane. Neuroserpin knockout mice (*Ns^−/−^*) have been described before [13], [14]. All mice have been backcrossed to C57Bl\6 mice for at least ten generations and littermates of genetically modified mice were used as controls. All animal procedures were performed in accordance with the institutional guidelines from the animal facility of the University Medical Centre Hamburg-Eppendorf. Before MCAO mice were recoded by an independent researcher to ensure necessary blinding. Sample size calculation was performed (stroke size from pilot experiments, significance level 0.05, power 90%.) and resulted in 10 animals per group to see a difference of 23% in stroke size respectively. For evaluation of infarct size at day three 10 wt and 10 *Ns^−/−^* mice were included into the experiment. In the *Ns^−/−^* group two mice were excluded since they reached dropout criteria. In the analysis of the survival rate until day seven 13 wt and 13 *Ns^−/−^* mice were included. An overview of further experimental groups is shown in **Fig. S1D**.

### In Vivo Stroke Model

Temporary middle cerebral artery occlusion (MCAO) was done as previously described [15]. MCAO was achieved by using the intraluminal filament method (6–0 nylon) for one hour. All mice (20 to 25 g, 12 weeks; TVH, University Medical Center Hamburg-Eppendorf) were anesthetized (isoflurane 1% to 2% v/v oxygen) and underwent analgesia (buprenorphine 0.03 mg/kg body weight intraperitoneally every 12 hours for 24 hours). All mice were monitored for rectal body temperature, and cerebral blood flow using transcranial temporal laser doppler (Moor Instruments, Millwey Axminster, UK). After stroke induction, every mouse was repeatedly scored on a scale from 0–5 (0 no deficit, 1 preferential turning, 2 circling, 3 longitudinal rolling, 4 no movement, 5 death) immediately after reawakening and every day until sacrifice. Rectal body temperature also showed no difference in our cohorts (data not shown). Mice were sacrificed one, three or seven days after reperfusion using isoflurane and decapitation. Only mice with a score greater or equal than one after reawakening were included for stroke size analysis, and only the animals with a visible cortical infarct were included for FACS analysis of infiltrating cells.

### Analysis of Infarct Size

For analysis of infarct size, brains were harvested, cut into 1 mm slices (Braintree Scientific, Braintree, USA) and vital staining using 2% (w/v) 2,3,5-triphenyl-2-hydroxy-tetrazolium chloride (TTC, Sigma-Aldrich, St Louis, USA) in phosphate buffer was performed. Infarct volumes were determined by blinded examiners using NIH ImageJ and statistics (T-test, Graph Pad Prism, La Jolla, USA).

### Antibodies and Flow Cytometry

Flow cytometry for the analysis of cell types was performed as previously described [15]. Mouse antibodies were as follows (all from eBioscience, San Diego, USA): CD45 (30-F11), TNF-α (MP6-XT22), Ly6G (1A8), CD11c (N418), CD11b (M170). For intracellular cytokine staining animals were euthanized and perfused with phosphate-buffered saline. Only ipsilesional hemispheres were dissected, digested for 30 min at 37°C (1 mg/ml collagenase, 0.1 mg/ml DNAse I in DMEM), and pressed through a cell strainer. Cells were incubated with standard erythrocyte lysis buffer on ice and separated from myelin and debris by Percoll gradient (GE Healthcare, Buckinghamshire, GB) centrifugation. For intracellular staining of microglia, intracellular transport was blocked with brefeldin A (3 µg/ml; eBioscience) for 3 hours. After staining of surface markers cells were fixed, permeabilized and stained for intracellular cytokines using (IC) Fixation Buffer in conjunction with Permeabilization Buffer (eBioscience). For absolute quantification, TrueCount tubes (BD Biosciences, Franklin Lakes, USA) containing fluorescence beads were used according to the manufacturer’s protocol and 10% of the cell volume was counted. Data were acquired with a LSR II FACS system (BD Biosciences) and analyzed with FlowJo (TreeStar, Ashland, USA). Doublets were excluded with FSC-A and FSC-H linearity.

### Immunohistochemistry

For histological analysis of mouse brains, animals were perfused with 4% buffered formalin and post-fixed over night. Sections were chosen in distance of about 2.5 to −3.5 mm from bregma. One picture of the dentate gyrus or the penumbra of each individual was taken and cells were counted from a blinded experimenter to reduce the risk of counting being biased. Brains were stained following standard immunohistochemistry procedures using the Ventana Benchmark XT (Ventana, Tuscon, USA). Briefly, deparaffinezed sections were boiled for 30–60 min in 10 mM citrate buffer (10 mM sodium citrate, 0.05% Tween20, pH 6.0), for antigen retrieval. Primary antibodies against GFAP (1∶200, DAKO, Hamburg, Germany), CD3 (1∶100, DAKO), Iba-1 (1∶2000, Wako, Neuss, Germany) and Ly6G clone 1A8 (1∶1000, Biolegend, San Diego, USA) were diluted in 5% goat serum, 45% Tris-buffered saline pH 7.6 (TBS) and 0.1% Triton X-100 in antibody diluent solution (Zytomed, Berlin, Germany) and incubated for 1 h. Anti-rabbit, anti-mouse or anti-goat histofine Simple Stain MAX Universal immunoperoxidase polymer (Nichirei Biosciences, Wedel, Germany) were used as secondary antibodies and detected with DAB solution. Sections were covered (Sakura Finetek, Staufen, Germany), dried and pictures were taken using a light microscope and digital camera (Zeiss, Jena, Germany). Immunofluorescence stainings of vessels were performed with an anti-CD31-antibody (1∶50, Dianova, Hamburg, Germany) according to standard protocols.

### Western Blot

For detection of fibrinogen and fibrin levels brain homogenates were prepared with RIPA-buffer containing 2% SDS, 1% NP-40, 150 mM NaCl and 25 mM Tris pH 7.4 and pulsed with ultra-sound. Alternatively, to avoid degradation of fibrin, brains were prefixed with 4% PFA and samples were boiled for 20 min at 90°C followed by 2 h at 60°C. After centrifugation for 30 min at 13,000 rpm at 4°C 20 µg protein from the supernatants were separated with a 12% SDS-PAGE and blottet on a PVDF membrane. Blots were incubated with primary polyclonal rabbit-anti-mouse antibody detecting fibrin and fibrinogen (1∶500, Acris antibodies, San Diego, USA) overnight at 4°C. After incubation with secondary anti-rabbit HRP coupled antibody, antigen-antibody reaction bands were visualized using a chemiluminescence detection kit (Thermo Scientific, Rockford, USA). The signals were visualized using Imager Gel Doc System and Quantity One software (Bio-Rad, Munich, Germany).

### Assessment of the Cerebral Vasculature

For assessment of the cerebral vasculature wt and *Ns^−/−^* mice (n = 3/group) were deeply anesthetized with CO_2_. Post mortem, mice were injected with an acrylic**-**based resin (Acryfix, Evonik, Dortmund, Germany), for preparation of anatomical corrosion castings. After hardening, tissues were corroded away with 25% KOH, and 1 removed carefully with forceps. The plastinated vasculature was exposed and examined under a binocular.

### Statistics

Data are reported as mean±SD. Statistical analyses were performed using the appropriate test indicated in the figure legends. Briefly, Student’s t test was used to compare infarct volumes; Mann–Whitney U test for the comparison of clinical scores, and one-way ANOVA for multiple comparisons with Bonferroni’s post-hoc test, after validating the normal distribution of these data sets (Kolmogorov–Smirnov test). *P* values <0.05 were considered statistically significant.

## Results

### Deficiency in Neuroserpin is Detrimental in Stroke

**Figure 1:**
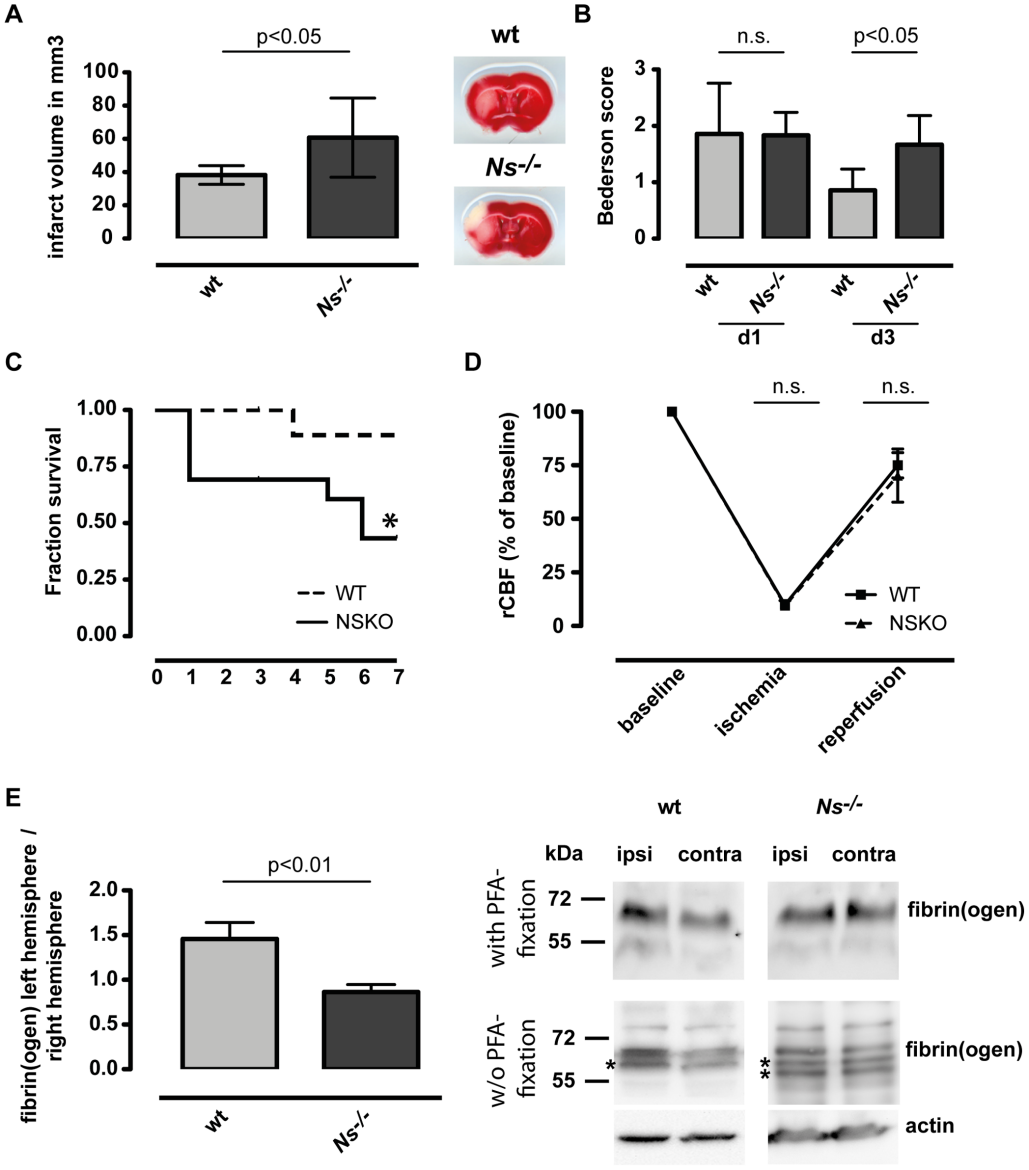
Deficiency in neuroserpin is detrimental in stroke. (**A**) TTC staining for evaluation of infarct volume at day three (left panel) and (**B**) neurological scores at days one and three (right panel) of wt and *Ns^−/−^* mice after MCAO. Data are represented as means±SD of 10 wt and eight *Ns^−/−^* animals. *t* test was used to assess statistical significance for infarct sizes and Mann-Whitney U test for neurological scores. (**C**) Survival rate of wt (n = 13) and *Ns^−/−^* mice (n = 13). Survival was analyzed by the *χ* ^2^ test (survival rate). (**D**) In all mice subjected to MCAO, regional cerebral blood flow (rCBF) was measured with Laser Doppler. The decrease in rCBF of approximately 90% was similar between *Ns^−/−^* and wt mice. Ten minutes after reperfusion rCBF was reconstituted to at least 60% of baseline levels and was unaltered between *Ns^−/−^* and wt animals. (**E**) Accumulation of fibrin(ogen) in the infarcted and in the contralesional hemispheres of wt (n = 3) and *Ns^−/−^* mice (n = 3). Fibrin(ogen) formation was analyzed by immunoblotting following fixation with 4% PFA (upper panel) or w/o 4% PFA-fixation (lower panel) using a rabbit polyclonal fibrin/fibrinogen-specific antibody 24 h following ischemia. Asterisks indicate additional bands representing fibrin degradation. *t* test was used to assess statistical significance.

Treatment with neuroserpin as well as its overexpression result in significantly decreased stroke volumes [5], [8]. To assess if the deficiency of neuroserpin leads to an increase in tissue damage following stroke, we analyzed infarct size and neurological scores in neuroserpin deficient (*Ns^−/−^*) mice and wt mice after 1 h MCAO. Three days after ischemia, *Ns^−/−^* mice showed a significantly increased infarct size compared to wild type animals from 38.3±5.6 mm^3^ to 61.0±23.8 mm^3^ (**Fig. 1A**). Also the neurological disability three days following cerebral ischemia was significantly worse in *Ns^−/−^* mice compared to wt mice (**Fig. 1B**). To asses whether the deficiency in neuroserpin goes along with a delayed regeneration at later time points, we followed mice up to day 7 after stroke and found a significantly reduced survival rate in *Ns^−/−^* mice (88,9% in wt mice vs 43,3 in *Ns^−/−^* mice **(** **Fig. 1C** **)**. To demonstrate that the differences in infarct size and survival between both strains was not determined by differences in the vasculature of the brain, we investigated (i) the cerebral blood flow by laser doppler before, during, and after occlusion **(** **Fig. 1D** **)**; (ii) the vascular anatomy of the Circle of Willis **(Fig. S1A)**; and (iii) the cortical mean vessel density **(Fig. S1C)**. None of the parameters, including the body weight of wt and *Ns^−/−^* mice before MCAO (**Fig. S1B**), showed any differences.

### Fibrin and Fibrinogen Levels are Decreased in Ischemic Hemisphere of in *Ns^−/−^* Mice

To study tPA activity in the presence or absence of neuroserpin, we determined levels of fibrin and fibrinogen, the latter being a direct substrate of plasminogen, in ischemic hemispheres of wt and *Ns^−/−^* mice. One day following MCAO we observed an increase of fibrin and fibrinogen in the ipsilateral hemisphere of wt mice when compared to their contralateral hemisphere. However, in *Ns^−/−^* mice no such difference could be detected between both hemisperes, indicating an increased tPA activity in the ischemic hemispheres of *Ns^−/−^* mice (**Fig. 1E**). Due to fibrin degradation additional Western blots were performed. To cross-link fibrin and devoid degradation, brains were pre-fixed with 4% PFA before protein preparation. In accordance with Western blots performed w/o fixation, we observed increased fibrin(ogen) levels in the wt on the ipsilateral side, whereas in *Ns^−/−^* mice no such difference could be detected (**Fig. 1E**).

### Deficiency in Neuroserpin does not Lead to Alterations of the Cellular Post Stroke Infiltrate

**Figure 2:**
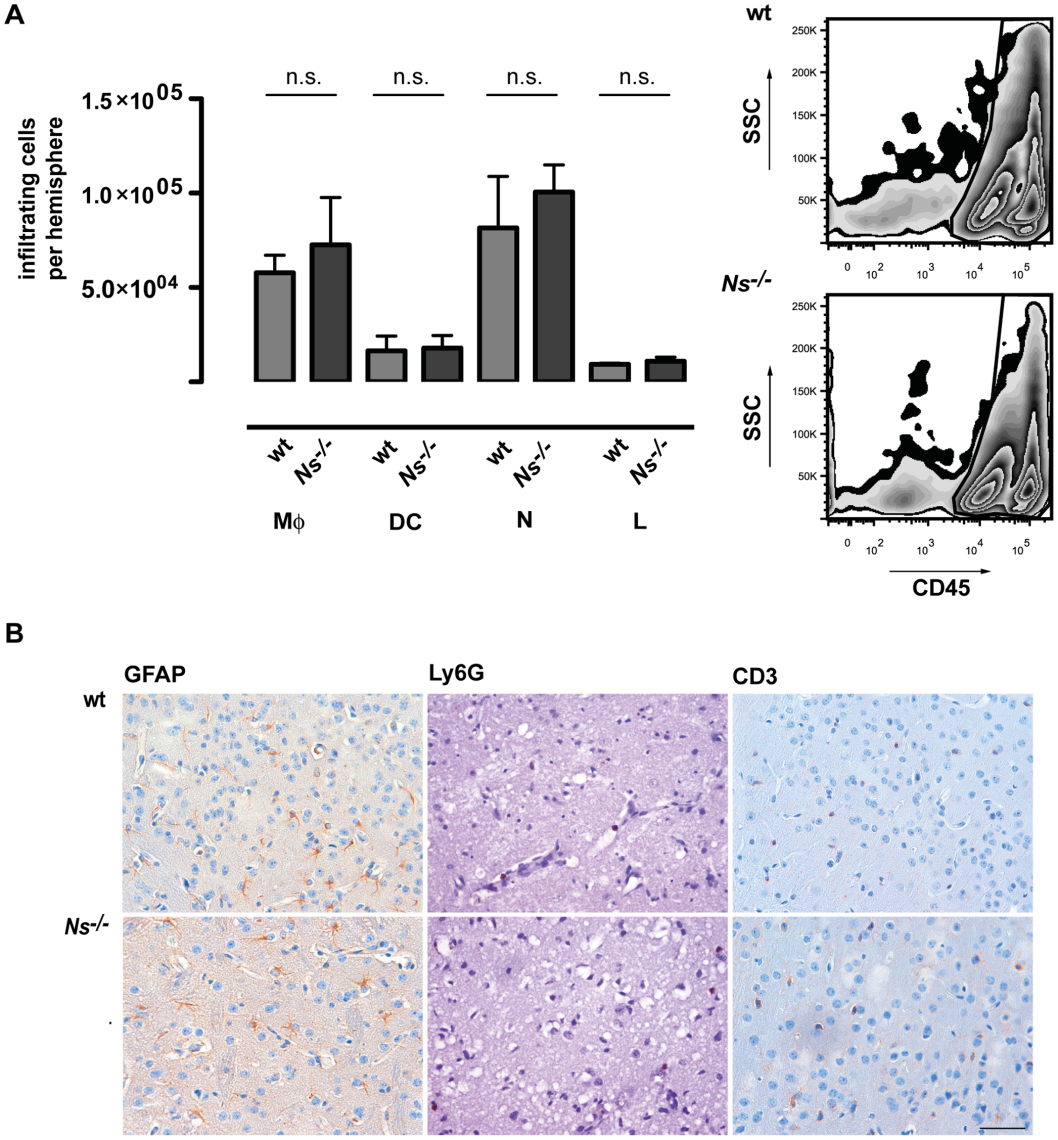
Deficiency in neuroserpin does not lead to alterations of the cellular post stroke infiltrate. (**A**) Absolute numbers of neutrophils (N), macrophages (Mφ), dendritic cells (DC) and lymphocytes (L) in the CNS-infiltrating cells of *wt and Ns^−/−^* mice. Representative plots are shown for CD45^high^ and CD45^intermediate^ cells. Brain macrophages were identified as CD45^high^, CD11b^+^, CD11c^−^ and distinguished from microglia by the higher expression of CD45. Dendritic cells were identified as CD45^high^, CD11b^+^, CD11c^+^, neutrophils as CD45^high^, CD11b^+^, Ly6G^+^ and lymphocytes as CD45^high^, CD11b^−^, CD11c^−^. The graphs show the means±SD of 9 animals per group analyzed three days after MCAO in three or four independent experiments. One-way ANOVA with Bonferroni post-hoc test was used to assess statistical significance. (**B**) Immunohistochemical staining of T cells (CD3), neutrophils (Ly6G) and astrocytes (GFAP) in wt and *Ns^−/−^* mice three days after MCAO (scale bar = 50 µm).

tPA has been shown to activate immune cells which could explain the larger infarcts [16]., We used whole brain FACS to analyze the composition of the infiltrating immune cells of the ischemic hemisphere of wt and *Ns^−/−^* mice 3 days following reperfusion. In both mice strains, we observed a cellular infiltrate dominated by CD45^high^/Ly6G^+^/CD11b^+^ neutrophils and CD45^high^/Ly6G^−/^CD11b^+^ macrophages followed by CD45^high^/CD11c^+^/CD11b^+^ dendritic cells and by CD45^+^/CD11b^−/^Ly6G^−^ lymphocytes (**Fig. 2A**). There was no statistical difference between wt and the *Ns^−/−^ mice*. The same was true for immunohistochemical analysis of infiltrating Ly6G positive neutrophils and CD3 positive T cells (**Fig. 2B**). There was also no difference in the response of astrocytes shown by immunohistochemical staining of the marker protein GFAP (**Fig. 2D**).

### Activation of Microglia is Increased in the Absence of Neuroserpin

Another important target of tPA could be microglial cells. Microglia proliferates in ischemic cerebral tissue and secretes pro-inflammatory mediators including TNF-α, ROS and IL-1β [17]. We examined the proliferation and activation of microglia in wt vs *Ns^−/−^* mice three days following MCAO.

**Figure 3:**
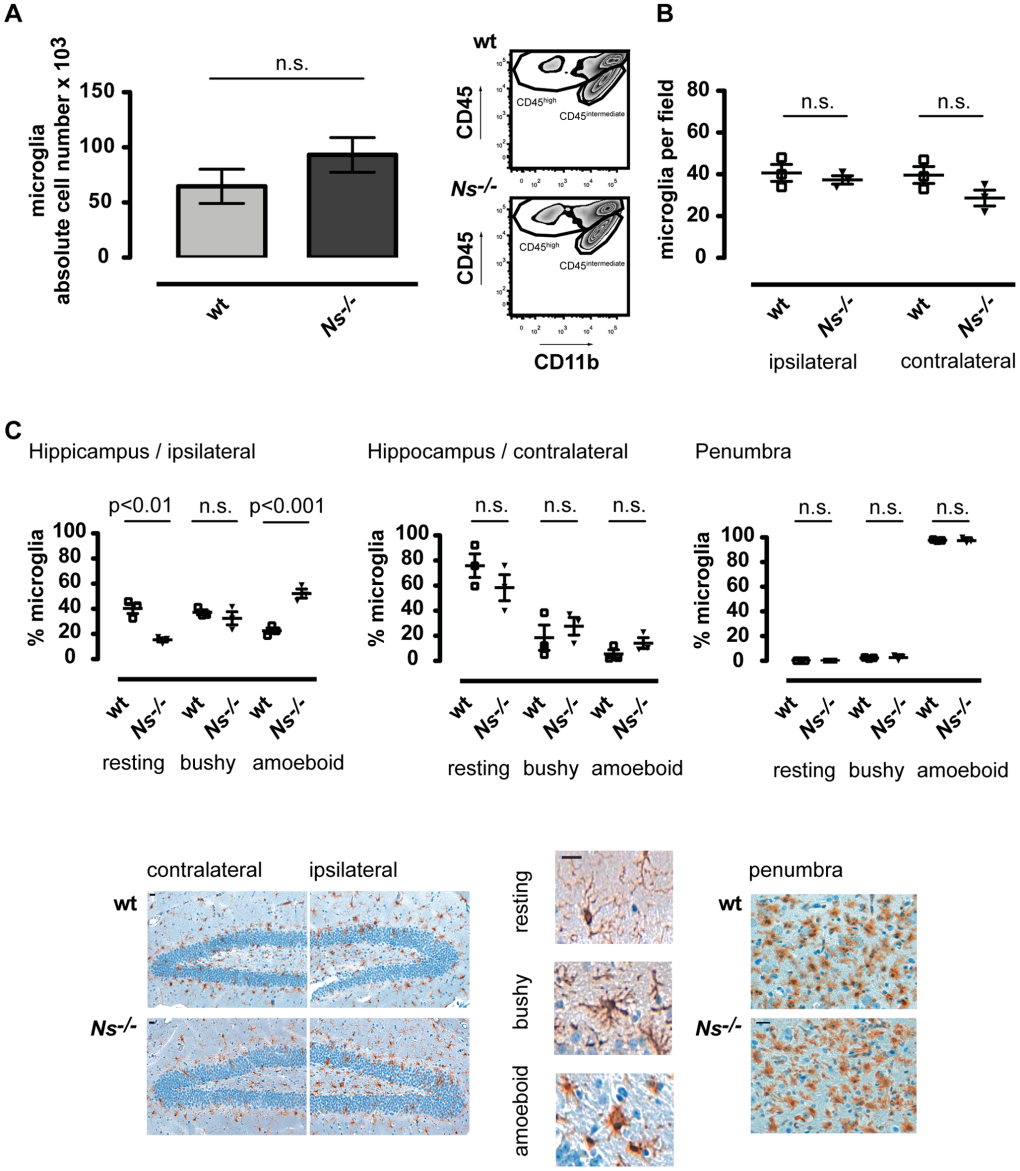
Activation of microglia is increased in the absence of neuroserpin. (**A**) Absolute numbers of brain microglia in the ischemic hemisphere of wild type, and *Ns^−/−^* mice 3 days after MCAO. Cell counts were determined by flow cytometry analysis of the CNS-infiltrating cells using TrueCount tubes. Brain microglia cells were identified as CD11b^+^ CD45^intermediate^. Representative dot plots show CD11b^+^ CD45^high^ and CD11b^+^ CD45^intermediate^-gated populations identifying macrophages and microglia respectively. The graphs show means±SD of 9–12 animals per group analyzed three days after MCAO in three or four independent experiments. *t* test was used to assess statistical significance. (**B**) Immunohistochemical analysis of absolute numbers of Iba-1 positive brain microglia/macrophages in the ischemic hemisphere of wild type, and *Ns^−/−^* mice 3 days after MCAO. The graphs show means±SD of 3 animals per group. *t* test was used to assess statistical significance. (**C**) Immunohistochemical analysis of the activation state (resting, bushy and amoeboid) of Iba-1 positive microglia in the ipsilesional hippocampus and penumbra area and contralesional hippocampus area 3 days following 1 h MCAO. The graphs show means±SD of 3 animals per group and the statistical analysis was assessed using one-way ANOVA with Bonferroni post hoc test (scale bar = 20 µm).

There was no statistical significant difference in the absolute number of CD45^intermediate^/CD11b^+^ microglia (**Fig. 3A**). This could be confirmed with immmunohistochemical studies of Iba-1 positive macrophages/microglia (**Fig. 3B**).

However, when differentiating activated (amoeboid) from resting (ramified) Iba-1 positive cells in the hippocampus 52,2±6.3% in the *Ns^−/−^* mice vs 22.6±3.9% in the wt animals had an activated phenotype and only 15.3±2.8% vs 40.2±7.0% exhibited a resting morphology (**Fig. 3C**). In the penumbra area 97% of the Iba-1 positive cells had an activated phenotype in both strains (**Fig. 3C**).

**Figure 4:**
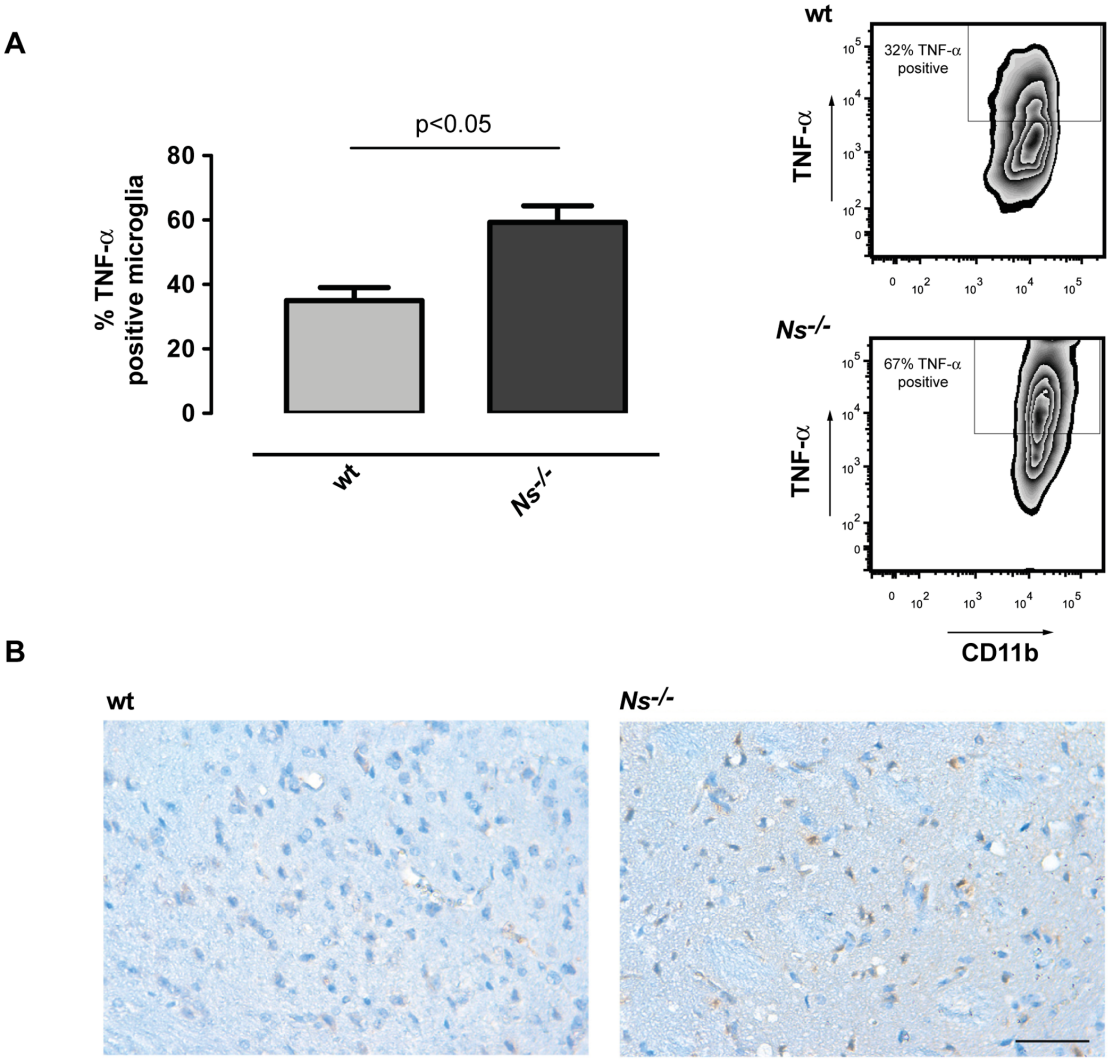
Activation of microglia is increased in the absence of neuroserpin. (**A**) Frequency of TNF-α positive brain microglia in wt and *Ns^−/−^* mice analyzed by flow cytometry three days after stroke. Representative dot plots show CD11b^+^ CD45^intermediate^-gated microglia. The graphs show means±SD of 9–12 animals per group analyzed three days after MCAO in three or four independent experiments. *t* test was used to assess statistical significance. (**B**) Immunohistochemical analysis of TNF-αexpression in ischemic hemispheres 3 days following 1 h MCAO showing an increased TNF-α immunoreactivity in ischemic tissue from *Ns^−/−^* mice compared to wt mice (scale bar = 50 µm).

We used TNF-α expression of microglia to determine microglial activation. While in wt mice 35.0±7.0% of the microglia were positive for TNF-α the amount of microglia producing TNF-α in *Ns^−/−^* mice was significant increased to 59.3±8.7% (**Fig. 4A**). The increased TNF-αlevels in *Ns^−/−^* mice could be confirmed in immunohistochemical studies, showing an increased abundance of TNF-α positive cells with microglial morphology in *Ns^−/−^* compared to wt mice (**Fig. 4B**).

Thus, *Ns^−/−^* mice suffer from greater tissue damage following MCAO, which correlates with an increase in activated, TNF-α producing microglia.

## Discussion

Neuroserpin, a neuron-specific serine protease inhibitor, is implicated in neuronal development, synaptic plasticity and it acts as a neuroprotective agent with plasminogen activators as target proteases [18]. In cerebral ischemia the rapid up-regulation of neuroserpin in the penumbra area [5], [8] has been suggested to represent an endogenous protective mechanism leading to neutralization of deleterious tPA effects. In fact, increased levels of neuroserpin following stroke are clinically associated with better functional outcome [10]. Endogenous tPA can be produced by microglia, neurons, and endothelial cells and is rapidly released in cerebral ischemia [19], [20] whereas recombinant tPA (rtPA) is commonly used as a thrombolytic agent to lyse blood clots. Detrimental tPA effects include neuronal excitotoxicity, blood brain barrier (BBB) breakdown, and activation of microglia. The importance of a balanced expression of tPA and neuroserpin could be demonstrated in transgenic mice models in which either the deficiency for tPA or the overexpression of neuroserpin were protective in ischemic stroke [3], [5], [8].

As expected, the lack of neuroserpin led to a significantly increased tPA activity in *Ns^−/−^* mice after stroke. Consistent with the previously described beneficial role of neuroserpin in cerebral ischemia, *Ns^−/−^* mice showed a significant increased infarct size and worse neurological outcome after MCAO at day 3. The similar neurological scores at day 1 is most likely due to the initial stroke-induced network disruption. However, after this point wt animal recover better since their lesion are smaller. In correspondence, the survival rate of *Ns^−/−^* mice was significantly reduced. The worse outcome in the *Ns^−/−^* mice occurred despite less thrombus formation and a presumably better reperfusion. Consequently detrimental tPA effects must have other reasons.

It has been shown that tPA can induce proinflammatory pathways in immune cells [20]. The observation that microglia is activated by tPA activity might represent one mechanism by which tPA leads to further progression of the ischemic damage [19], [21]. Fitting this hypothesis, we observed significant changes in the activation profile of microglia including up regulation of TNF-α in the absence of neuroserpin. The differences in microglia activation were marked in remote areas of the ipsilateral hemisphere related to the infarct core, whereas in direct proximity nearly 100% of the microglia were activated in both strains. This finding indicates that a normal complete activation of microglia occurs in the penumbra areas, whereas the activation in remote areas is influenced by additional stimuli including tPA. In stroke, microglia already exert neurotoxic effects via release of superoxide [22], nitric oxide [23], TNF-α, glutamate [24] and metalloproteinases [25] resulting in direct cytotoxic effects and blood brain barrier damage [26] which are further boosted by tPA.

Possible mechanisms of microglia activation by tPA include non-proteolytic pathways [16]. The signaling seems to be dependent on LRP1, a member of the Low-density-lipoprotein-receptor gene family. LRP1 is expressed on neurons, microglia, and perivascular astrocytes and interaction of tPA with LRP1 is detrimental in stroke [21], [27]. The exposure of microglia to tPA is possibly enhanced by paracrine and autocrine feedback loops [19].

It is important to realize that microglia has also anti-inflammatory and neuroprotective properties [28]. In stroke microglia activation is associated with tissue repair and regeneration. Mechanisms include phagocytosis of debris, remodeling of the extracellular matrix, release of immunmodulatory cytokines/trophic factors [28], [29], and direct engulfment of neurotoxic neutrophils [30].

The multifaceted effects of tPA are of high interest, since clinical studies have shown deleterious effects in patients treated with rtPA for thrombolysis despite the unquestionable beneficial results in large clinical trials. Large intracranial hemorrhages were seen in 5.2% in the treatment group and in 1.0% of the controls [31], which could be a result of increased microglia derived matrix metalloproteinases in the presence of exogenous tPA [32]. One potential extension of our study could have been the use an embolic stroke model. However, there are several drawbacks to this model. The downsides of embolic models are that they are hard to perform in mice, they have a bigger variance in stroke sizes, and they would make it difficult to distinguish between positive and negative tPA effects.

The effects of tPA on microglia suggest that a combined treatment with anti-inflammatory drugs might be a worthwhile consideration. In summary, our data supports the protective role of neuroserpin in cerebral ischemia and indicates that the unbalanced expression of neuroserpin and tPA in *Ns^−/−^* mice leads to worse outcome in experimental stroke, which is associated with increased microglia activation.

## Supporting Information

Figure S1 Characterization of the cerebral vasculature in *Ns^−/−^* and wt mice. Cerebral vasculature was assessed macroscopically and in immune fluorescence. **(A)** Plastinates of the cerebral vasculature of wt (blue) and *Ns^−/−^* mice (red) revealed an intact Circle of Willis in all animals (n = 3 per group), and the distribution of the MCA trunk and branch appeared to be anatomically unaltered in the different genotypes (asterisks). **(B)** Body weight before MCAO was unaltered between wt and *Ns^−/−^* mice. **(C)** Additionally, histological analyses showed similar cortical mean vessel density. Vessels were stained with anti-CD31-antibodies shown in red; nuclei were stained with DAPI; scale bar 100 µm. Binding of the primary antibodies was visualized after incubation with the appropriate fluorescently labeled secondary antibodies. Microvessel densities were 444.7±39 vessels/mm^2^ vs. 454.4±51 vessels/mm^2^ in wt and *Ns^−/−^* mice, respectively. **(D)** Experimental groups and number of wt and *Ns^−/−^* mice entered into the study.
(TIF)
