# Supplementary figures and images for: Deficiency in Serine Protease Inhibitor Neuroserpin Exacerbates Ischemic Brain Injury by Increased Postischemic Inflammation
Source: PLoS One. 2013 May 3;8(5):e63118. doi: 10.1371/journal.pone.0063118 (PMC3643909; doi:10.1371/journal.pone.0063118)

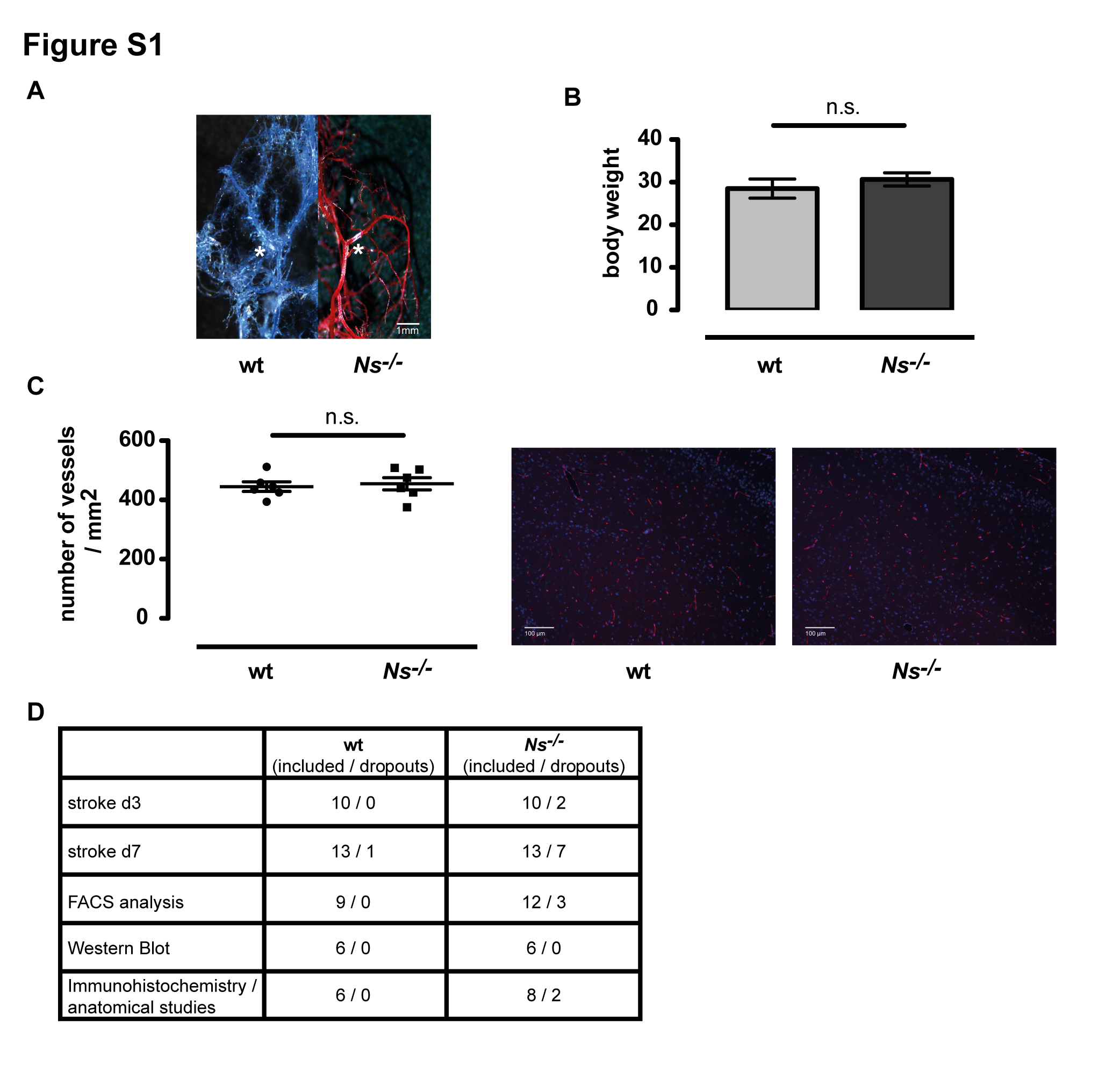

Supplement: Figure S1 — Characterization of the cerebral vasculature in Ns−/− and wt mice. Cerebral vasculature was assessed macroscopically and in immune fluorescence. (A) Plastinates of the cerebral vasculature of wt (blue) and Ns−/− mice (red) revealed an intact Circle of Willis in all animals (n = 3 per group), and the distribution of the MCA trunk and branch appeared to be anatomically unaltered in the different genotypes (asterisks). (B) Body weight before MCAO was unaltered between wt and Ns−/− mice. (C) Additionally, histological analyses showed similar cortical mean vessel density. Vessels were stained with anti-CD31-antibodies shown in red; nuclei were stained with DAPI; scale bar 100 µm. Binding of the primary antibodies was visualized after incubation with the appropriate fluorescently labeled secondary antibodies. Microvessel densities were 444.7±39 vessels/mm2 vs. 454.4±51 vessels/mm2 in wt and Ns−/− mice, respectively. (D) Experimental groups and number of wt and Ns−/− mice entered into the study. (TIF) [file pone.0063118.s001.tif]
